# Clinical Features of Intestinal Ulcers Complicated by Epstein-Barr Virus Infection: Importance of Active Infection

**DOI:** 10.1155/2021/6627620

**Published:** 2021-05-03

**Authors:** Yuyuan Liu, Yuqin Li, Yajun Li, Shuang Wu, Xinyue Tian, Tongyu Tang, Haibo Sun, Chuan He

**Affiliations:** ^1^Department of Gastroenterology, Bethune First Affiliated Hospital of Jilin University, Changchun, Jilin, China; ^2^Department of Gastroenterology, Affiliated Hospital of Shandong Medical College, Changchun, Jilin, China

## Abstract

Clinical characteristics of intestinal ulcers complicated with Epstein-Barr virus (EBV) infection remain poorly studied. This study is aimed at providing further insight into clinical features of this patient cohort. The presence of serum EBV DNA was assessed in 399 patients with colonic ulcers, of which 30 cases were positive. In EBV-positive patients, the EBV-encoded RNA (EBER) was detected in intestinal tissues of 13 patients (EBER-positive group). The test was negative in 17 patients (EBER-negative group). Acute EBV infection rate in patients with colonic ulcer was 7.52%. Age and sex differences between two groups were not statistically significant. Fever, abdominal lymph node enlargement, and crater-like gouged ulcer morphology were more common in the EBER-positive group (*P* < 0.05). The albumin level in the EBER-positive group was significantly lower compared to that in the EBER-negative group (*P* < 0.05). The copy count of EBV DNA in the blood of patients from the EBER-positive group was higher, and the prognosis was worse (*P* < 0.05). Clinical manifestations were more severe in the EBER-positive group. Endoscopic, histopathological, and biochemical findings were also more serious in this group of patients. The findings point to the importance of assessing the EBER expression in patients with intestinal ulcers of various etiology. EBER positivity should be viewed as a diagnostic marker of more severe condition requiring more aggressive treatment.

## 1. Introduction

Epstein-Barr virus (EBV), also known as human herpesvirus-4 (HHV-4), is a double-stranded DNA virus of the herpesvirus family [1]. Primary EBV infection usually occurs in childhood. More than 90% of adults have EBV infection which persists for a lifetime. Most of these infections are self-limited or cause infectious mononucleosis (IM) [2]. The infection may be linked to more serious conditions, such as chronic active EBV infection (chronic active EBV (CAEBV)), EBV-positive lymphoproliferative disease (LPD), and related tumors [3], or participate in the occurrence and development of a variety of autoimmune diseases [4]. Intestinal EBV infection can be manifested by nonspecific symptoms such as fever, hematochezia, abdominal pain, and diarrhea, which are difficult to distinguish from inflammatory bowel disease (IBD) and can be easily misdiagnosed, with serious consequences. EBV infection makes people more likely to develop IBD, which may lead to persistent inflammation and inadequate response to conventional treatment. This, in turn, has a potential effect on refractory IBD. IBD increases the risk of intestinal EBV infection [5–7].

EBV infection in the intestinal mucosa and its role in the progression and deterioration of intestinal diseases attracted wide attention. However, there is a lack of clear diagnostic criteria and treatment for EBV infection-related bowel disease. Therefore, this study is aimed, through the analysis and discussion of the clinical characteristics of EBV-related bowel disease, to increase clinicians' understanding of the condition, provide a basis for its early diagnosis and treatment, and improve the prognosis.

Currently, there are only few reports about EBV infection with gastrointestinal manifestations as the main symptoms. Most of these publications are either case reports or research reports, and they analyze the clinical characteristics of chronic EBV infection-associated enteritis [8, 9]. This is the first study of the clinical characteristics of intestinal ulcers complicated with acute EBV infection.

We summarized 30 cases of colorectal ulcers with positive blood EBV DNA test results. We described their clinical symptoms, endoscopic manifestations, treatment, and prognosis, with special focus on the characteristics of EBER-positive patients.

## 2. Materials and Methods

This study collected the data of 399 patients with colorectal ulcers treated in the First Hospital of Jilin University from October 2016 to October 2018 (24 patients with intestinal tuberculosis, 275 patients with IBD, 45 patients with ischemic bowel disease, and 55 patients with other conditions). PCR was used to detect serum EBV-DNA, with the DNA copy number greater than 500 copies/ml considered positive. Thirty cases were identified as positive for EBV (18 patients with ulcerative colitis, 3 patients with Crohn's disease, 1 patient with chronic EBV-associated lymphoproliferative disease, 4 patients with chronic active EBV infection, and 4 patients with ulcers of unknown origin). After excluding the patients with incomplete laboratory data, those without blood EBV DNA test, those without the results of enteroscopic examination, and those diagnosed with intestinal lymphomas, the detailed clinical data of 30 hospitalized patients with colorectal ulcers complicated by EB viremia were analyzed retrospectively.

Tissues from colon ulcer sites were evaluated by biopsy and *in situ* hybridization. Based on the test results, the patients were divided into the EBER-positive group and EBER-negative group. EBER *in situ* hybridization is considered the gold standard assay for detecting the latent EBV infection.

Mucosa for *in situ* hybridization (ISH) was biopsied from areas near the inflamed areas, fixed in formalin immediately, and embedded in paraffin blocks. ISH was conducted with EBER ISH Kit (ZSGB-BIO, Ltd., Beijing, China). The sections were prepared, deparaffinated with xylene for 10 min, rehydrated with anhydrous alcohol for 5 min, digested by gastric enzyme for 30 min, and incubated at 37°C overnight with hybridization solution containing the EBER-probe. After washing with PBS, the signal was amplified using anti-biotin antibody. A tissue was considered EBER-positive if EBER signal was seen in nuclei.

Available data about patients (clinical manifestations, results of laboratory tests, endoscopic manifestations, results of nutritional risk screening, administered treatments, and outcomes) were analyzed, and the comparison between two groups was made. The statistical software SPSS 20.0 was used for the analysis. Intergroup comparison was done using either independent sample *t*-test (normally distributed data) or rank sum test (not normally distributed data). The chi-square test or Fisher exact probability method was used for qualitative comparison of data. *P* < 0.05 was considered statistically significant.

## 3. Results

### 3.1. Sex Distribution

In the EBER-positive group, seven patients were males and six patients were females (the ratio is 1.17 : 1). In the EBER-negative group, there were 9 male and 8 female patients (the ratio is 1.125 : 1). There was no significant difference in the sex ratios between the two groups (*P* > 0.05), and there was no significant difference in the incidence of disease among males and females, as shown in Table 1S.

### 3.2. Age Distribution

In the EBER-positive group, five patients were in the youth group (18-44 years old), two patients in the middle-aged group (45-59 years old), and six patients in the elderly group (≥60 years old). In the EBER-negative group, five patients were in the youth group (18-44 years old), six patients in the middle-aged group (45-59 years old), and six patients in the elderly group (≥60 years old). There was no significant difference in the age distribution between the two groups (*P* > 0.05), as shown in Table 2S.

### 3.3. Clinical Manifestations

In both groups, the main clinical manifestations included abdominal pain, diarrhea, blood in the stool, and fever, while abdominal distension, vomiting, fatigue, and abdominal lymph node enlargement were rare. The clinical manifestations were similar between the two groups, with fever and abdominal lymph node enlargement more common in the EBER-positive group. The difference in the frequency of these two clinical manifestations was statistically significant (*P* < 0.05), as shown in Table 1.

### 3.4. Medical History

There was one previous case of autoimmune disease in the EBER-positive group, with no history of immune-related medication use. Three patients in the EBER-negative group had previous autoimmune diseases and had a history of immune-related medication use, and two patients were treated with infliximab. No statistically significant difference between the two groups was found (P >0.05), as shown in Table 3S.

### 3.5. Microscopic Assessment of Ulcers

**Figure 1:**
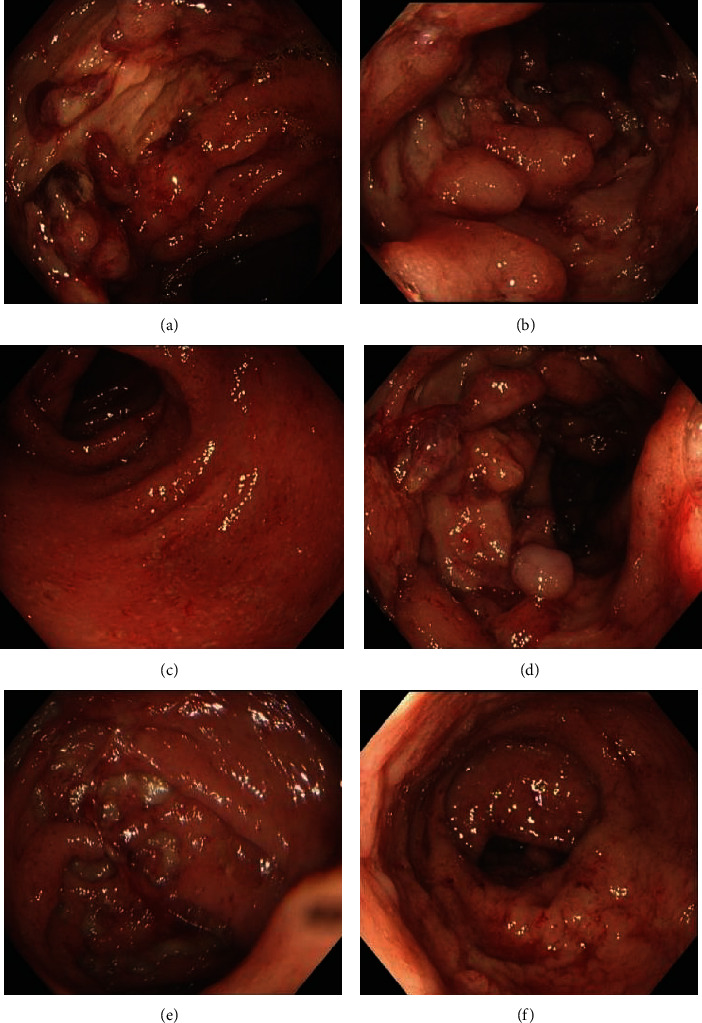
Endoscopic findings in patients with ulcerative colitis complicated with intestinal EBV infection ((a, b) ascending colon, (c) transverse colon, (d) descending colon, (e) sigmoid colon, and (f) rectum).

**Figure 2:**
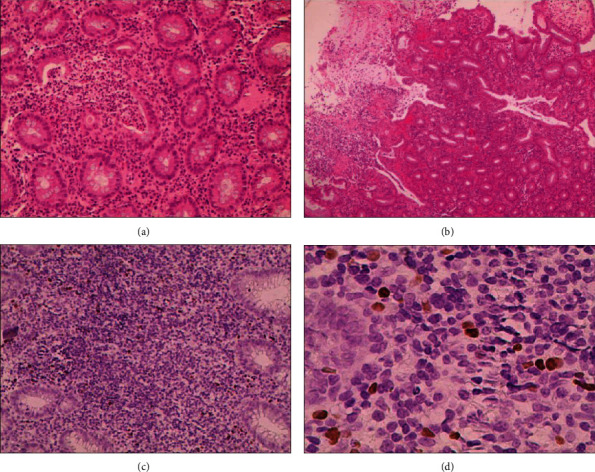
Histopathology of ulcerative colitis in patients with intestinal EBV infection: (a) magnification 200x; (b) 40x; HE staining. The mucosa of large intestine showed active chronic colitis, with fossa inflammation, fossa thickening, and local ulcer formation. (c) 200x; (d) 400x. EBER demonstrated EBV-positive lymphocytes of 40/HPF.

The morphology of colorectal ulcers in EBER-positive group showed multiple scattered depressions, chisel, irregular, round, and oval deep ulcerations, accompanied by mucosal congestion, edema, erosion, and infiltration. The ulcers in the EBER-negative group were mainly superficial, and there was a significant difference between the two groups (*P* < 0.05), as shown in Table 2. Figures 1 and 2 show endoscopic findings, histopathology, and EBER *in situ* hybridization results in patients with ulcerative colitis complicated with intestinal EBV infection.

### 3.6. Laboratory Tests

Albumin (ALB) was decreased in both groups. In both groups, some patients had one or two test abnormalities such as decreased peripheral blood cells count, increased inflammatory indicators, abnormal coagulation function, nonsignificant increase in liver function transaminase, decreased serum potassium, and increased EBV nucleic acid quantification, but the differences between the two groups were not statistically significant (*P* > 0.05), as shown in Table 3.

### 3.7. Nutritional Risk Screening Scores

The nutritional risk was assessed according to the nutritional risk screening score (NRS2002) of inpatients within 24 h after admission. We scored patients' nutritional status (based on weight loss > 5%, body mass index (BMI) < 18.5, and reduced food intake within a week) and disease severity on a score of 0 to 3 (0—no serious disease, 1—mild, 2—moderate, and 3—severe) and added one point for patients over the age of 70. The total score of NRS ≥ 3 indicates the risk of malnutrition [10]. The results of nutritional risk screening in both groups showed that nutritional risk might occur, and there was no significant difference between the two groups, but the proportion of nutritional risk in the EBER positive group was higher than that in the negative group, as shown in Table 4S. The relatively small size of research sample in this study may explain the lack of statistically significant differences.

### 3.8. Treatment Methods

Antiviral therapy included ganciclovir (0.5 g, 2/day) and hormone therapy-methylprednisolone (60 mg, 1/day). All of these patients received antiviral therapy, with the exception of 3 cases of self-healing. Most patients received either antiviral therapy, a combination of antiviral and hormone therapy, or a combination of antiviral and hormone therapy and additional surgical treatment. There was no significant difference between the two groups, but this may reflect a small sample size in this study. However, it can be seen from the data in Table 4 that patients with EBER-positive intestinal pathological tissue needed more hormonal and surgical treatments.

### 3.9. Clinical Outcomes

Improvement was defined as the improvement of clinical symptoms and the negative results of the EBV DNA blood test within 3 months. Improvements were observed in 6 patients (46.2% of patients) from the EBV-positive group and in 15 patients from the EBER-negative group (88.2% of patients). The difference was statistically significant (*P* < 0.05, Table 5), and the prognosis for the EBER-positive patients was worse than that for the EBER-negative patients.

## 4. Discussion

Epstein-Barr virus can affect the gastrointestinal tract causing ulcers and bleeding, even though this is not a frequent occurrence. EBV infections with gastrointestinal symptoms as the main manifestation are rare. Among these infections, the EBV infections with normal immune function and gastrointestinal manifestations are even more uncommon, and they are mostly reported as individual cases.

In this study, there were 399 patients with colorectal ulcers, and we tested them for serum EBV-DNA load, quantified intestinal mucosal EBER, and performed immunohistochemical evaluation. In this cohort, 30 patients were EBV seropositive, 17 patients were both EBV seropositive and EBER positive, 13 patients were EBV positive but EBER negative, and no patients with serum EBV-negative tests but tissue-EBER positive tests were found. The EBV RNA was mainly expressed in lymphocytes in the descending colon, rectum, and sigmoid colon. Virus expression in epithelial cells was not observed. The infection rate (measured by the presence of EBV in blood) was 7.52% in patients with colorectal ulcers and 7.64% in patients with IBD. In a Spanish study of 1483 adult patients with IBD, the seroprevalence of EBV was 97.4%, suggesting that patients with IBD had a higher past EBV infection rate [11]. In our experiments, the blood level of EBV DNA was measured. This is a more accurate approach to identify the current infection with EBV. Among all EBV-DNA positive patients, 13 cases were EBER positive (43.3%), and 17 cases were EBER negative (56.7%). Among 30 patients with EBV-related intestinal ulcers, 21 were diagnosed with IBD, which included 18 cases of ulcerative colitis and 3 cases of Crohn's disease. There were 7 cases of intestinal EBV infection in patients with IBD, with an EBER-positive rate of 33.3%. These findings were consistent with the study conducted in the Chinese population previously [6].

Among patients with IBD, males had a reduced past risk of EBV infection [11]. In this study, three of the four chronic active EBV- (CAEBV-) associated bowel disease patients were men. Of the 6 CAEBV-associated bowel disease patients, 4 were also males. The higher incidence of CAEBV in men is consistent with the previously published observations [9].

It was reported that age is a risk factor of intestinal mucosal EBV infection in patients with IBD in China [6]. In addition, the age over 30 years and smoking are also the risk factors of EBV infection in the IBD patients [11]. In this study, there was no significant difference in age distribution between the EBER-positive and EBER-negative groups, which contradicted the published findings cited above. The difference may be explained by the small sample size in our study. However, we found that intestinal ulcers associated with EBV infection might be more likely to occur in patients over the age of 60, perhaps because of weakened immunity in this age group. However, this question needs to be studied further on a larger cohort of patients.

The main clinical manifestations of EBV infection involving digestive tract are abdominal pain, diarrhea, and fever, accompanied by nonspecific manifestations such as weight loss and loss of appetite. The disease is characterized by progressive aggravation, and the prognosis of patients with hematochezia symptoms may be poor [8]. It is worth noting that these clinical symptoms are easily confused with IBD [12]. In this study, fever and celiac lymph node enlargement were more common in patients with EBV-related intestinal ulcers. Persistent high fever, persistent diarrhea, hepatomegaly, splenomegaly, and lymph node enlargement cannot be explained by IBD alone. The possibility of EBV infection should be considered, and detection and pathological examination related to EBV infection should be carried out in order to detect this opportunistic infection as soon as possible [13]. In this study, a patient with chronic EBV-LPD developed severe intestinal bleeding, complications, and hemorrhagic shock more than 2 months after the onset. In the past two years, most of the cases of EBV-LPD with normal intestinal immune function were misdiagnosed as IBD because of its similar clinical and endoscopic manifestations, resulting in poor prognosis or death [14–19]. Consistent with our case, these patients also developed intermittent high fever of unknown origin, characterized by recurrent periods of moderate to high fever, which subsided within 24 h [15]. Therefore, when intermittent high fever is observed in patients with intestinal ulcer of unknown cause, the possibility of EBV-LPD should be considered.

EBV is an opportunistic pathogen, which often affects elderly patients with weakened immune function or congenital and acquired immune deficiency. Gastrointestinal involvement rarely shows the tendency to canceration [17]. In recent years, there were also reports about EBV-LPD in patients with normal immune function, most of which were related to the use of certain drugs to treat IBD [20]. In our study, however, the patients from this subgroup were not significantly different, which may be due to the small sample size. The proportion of smokers in the EBER-positive group is higher in our study, which may be due to the fact that smoking is a risk factor for EBV infection in IBD patients [11].

The main endoscopic manifestation of EBV infection is extensive colorectal ulceration that may involve the whole digestive tract, with the colon being the most common site [8, 9]. The main manifestations of CAEBV- associated bowel disease are multiple colorectal ulcers of various forms, such as scattered deep ulcers or shallow small ulcers, accompanied by mucosal erosion, hyperemia and edema, as well as intestinal stenosis in a few cases [8–10]. In this study, the EBV infection-related ulcers showed sunken, trenched, irregular, round, oval, and other deep ulceration accompanied by mucosal congestion, edema, erosion, and infiltration, without cobblestone. Longitudinal ulcers were observed in patients with Crohn's disease, continuous superficial granular ulcers, in patients with ulcerative colitis, or circular ulcers, in patients with intestinal tuberculosis [6, 9]. Therefore, when there are deep ulcers, multiple ulcers, and spontaneous bleeding, the possibility of EBV infection should be considered. It has also been reported [19] that atypical infiltration of intestinal mucosa infected by EBV can be used as a marker for EBV detection in patients with IBD.

Compared with the EBER-negative group, the EBER-positive group showed more obvious ulcers under microscopic examination (*P* < 0.05), which indicated that the EBER-positive group had more serious malnutrition problems. In our study, patients with EBV infection-related intestinal ulcers were more likely to develop hypoproteinemia and malnutrition, which is consistent with the majority of previous studies [21]. Both the EBER-positive group and the EBER-negative group have nutritional risk, but due to the small sample size in this study, there was no significant difference in the incidence between the two groups, although the proportion of nutritional risk in the EBER-positive group was higher.

This study compared the treatment methods of patients with intestinal EBV infection and those with nonintestinal EBV infection. Although there was no statistical difference between the treatment methods used in the two groups, the intestinal EBV infection led to a more serious condition, which resulted in the use of more active and diverse treatments. In this study, 3 patients with intestinal ulcer complicated with EBV were self-cured. We considered that the EBV infection in these 3 patients was a transient viral infection, which was self-limited due to either the absence or the improvement in the treatment of primary underlying intestinal disease, and thus, there was no need for specific therapeutic interventions. The treatment of patients with EBV-LPD lacks standard approach, and antiviral drugs have no definite effect since EBV is in a state of latent infection and is not sensitive to the drug-activated kinases [22]. Antiviral drugs, immunomodulators, and cytotoxic drugs have been tried in the treatment of CAEBV, but most of them achieved only a short-term temporary relief of symptoms, and there were no reports of long-term curative effect. Hematopoietic stem cell transplantation is currently considered the only curative treatment [21, 23].

Most adults with the EBV infection have a good prognosis, and only few patients develop malignant diseases with complex clinical manifestations, difficult diagnosis and treatment, and poor prognosis. CAEBV without stem cell transplantation has a poor prognosis, and patients may die of liver failure, HLH, or T-cell lymphoma [24]. Gastrointestinal autoimmune diseases due to the decline of immune function and the use of some drugs promote intestinal EBV infection that can further aggravate the primary disease. In this study, only 46.2% of patients with intestinal EBV infection-related intestinal ulcers experienced clinical improvement, as compared to the negative group, and one patient with EBV-LPD died of severe gastrointestinal bleeding. It can be seen that both treatment effect and prognosis of patients with EBV infection-related bowel disease remain poor.

For this reason, clinicians should consider the possibility of active EBV infection in the intestinal ulcer patients with unexplained high fever, sudden aggravation of clinical symptoms, and endoscopic ulcers. Correct early diagnosis and appropriate treatment of the disease can improve the prognosis.

The limitation of this study lies in the small number of cases resulting in the lack of statistical significance. Thus, many of our findings should be treated only as observations.

## 5. Conclusions

Fever and celiac lymph node enlargement are more prevalent in patients with intestinal EBV infection. Endoscopic ulcers in these patients are deeper than those observed in noninfected patients and mainly characterized by bullet pit-like and chisel-like changes. Hypoalbuminemia, nutritional risk, and malnutrition are also more common in this group of patients. Nutritional intervention, hormone, and surgical treatment are more commonly needed, and the treatment and prognosis are worse compared to uninfected patients.

Our findings point to the importance of assessing the EBER expression in patients with intestinal ulcers of various etiology. EBER positivity should be viewed as a diagnostic marker of more severe condition requiring more aggressive intervention.

## Figures and Tables

**Table 1:** Comparison of clinical manifestations between the groups (*n* (%)).

Clinical manifestations	EBER-positive (*n* = 13)	EBER-negative (*n* = 17)	*χ* ^2^	*P* values
Abdominal pain	10 (76.9)	13 (76.5)	0.001	0.977
Diarrhea	11 (84.6)	14 (82.4)	0.027	0.869
Abdominal distension	4 (30.8)	6 (35.3)	0.068	0.794
Blood in the stool	10 (76.9)	11 (64.7)	0.524	0.469
Vomiting	2 (15.4)	3 (17.6)	0.027	0.869
Fever	9 (69.3)	4 (23.5)	6.266	0.012
Fatigue	3 (23.1)	4 (23.5)	0.001	0.977
Abdominal lymph node enlargement	6 (46.2)	2 (11.8)	4.455	0.035

**Table 2:** Endoscopic comparison of ulcer morphology between the two groups (*n* (%)).

Ulcer morphology		EBER-positive (*n* = 13)	EBER-negative (*n* = 17)	*χ* ^2^	*P* values
Morphology	Deep ulcerations	8 (61.5)	2 (11.8)	8.213	0.004
	Superficial ulcerations	5 (38.5)	15 (88.2)		

**Table 3:** Comparison of laboratory test results between the two groups.

Test	EBER-positive (*n* = 13)	EBER-negative (*n* = 17)	*t* values	*P* values
WBC (×10^9^/L)	6.50 (5.63, 8.43)	5.91 (4.08, 7.57)	-1.005	0.32
HB (g/L)	107.31 ± 23.47	98.58 ± 34.82	-0.777	0.444
PLT (×10^9^/L)	321.31 ± 149.67	305.71 ± 115.41	-0.323	0.749
ESR (mm/h)	20.0 (6.75, 49.0)	33.5 (13.0, 52.0)	-0.651	0.537
CRP (mg/L)	55.47 ± 44.09	50.14 ± 45.54	-0.318	0.753
PTA (%)	88.69 ± 21.82	95.25 ± 18.36	0.879	0.387
AST (U/L)	18.2 (16.85, 24.90)	18.7 (14.45, 28.35)	-0.251	0.802
ALT (U/L)	13.9 (8.55, 28.50)	13.3 (9.10, 23.75)	-0.356	0.722
GGT (U/L)	28.8 (14.45, 57.55)	17.3 (11.7, 38.5)	-0.942	0.346
ALP (U/L)	73.5 (53.95, 106.25)	60.8 (53.35, 99.90)	-0.649	0.516
CHE (U/L)	3470.23 ± 2502.58	4053.82 ± 2194.51	0.679	0.502
ALB (g/L)	22.38 ± 4.43	29.58 ± 7.33	3.121	0.004
K^+^ (mmol/L)	3.63 ± 0.83	3.55 ± 0.55	0.097	0.768
Cr (*μ*mol/L)	48.2 (38.1, 60.7)	62.7 (44.9, 78.2)	-0.148	0.137
EBV DNA (×10^3^ copies/ml)	12.3 (3.5, 25.6)	4.33 (1.52, 9.81)	-1.716	0.086

**Table 4:** Comparison of treatment methods between the two groups (*n* (%)).

Treatment method	EBER-positive (*n* = 13)	EBER-negative (*n* = 17)	*χ* ^2^	*P* values
Self-healing	1 (7.7)	2 (11.8)	5.059	0.168
Antiviral therapy	3 (23.1)	10 (58.8)		
Antiviral+hormone therapy	6 (46.2)	4 (23.5)		
Antiviral+hormone+surgical treatment	3 (23.1)	1 (5.9)		

**Table 5:** Clinical outcomes of patients in both groups (*n* (%)).

Outcomes	EBER-positive (*n* = 13)	EBER-negative (*n* = 17)	*χ* ^2^	*P* values
No improvements	7 (53.8)	2 (11.8)	6.212	0.013
Improvements	6 (46.2)	15 (88.2)		

## Data Availability

Data available from authors upon request.

## Supplementary Materials

Supplementary Materials Supplementary materials Table 1S: sex distribution of patients in two groups. Table 2S: age distribution of patients in both groups. Table 3S: medical history of patients from two groups. Table 4S: comparison of nutritional risk screening scores between two groups.
