# Supplementary material for: Clinical Features of Intestinal Ulcers Complicated by Epstein-Barr Virus Infection: Importance of Active Infection
Source: Dis Markers. 2021 May 3;2021:6627620. doi: 10.1155/2021/6627620 (PMC8110392; doi:10.1155/2021/6627620)
Supplement: Supplementary Materials — Table 1S: sex distribution of patients in two groups. Table 2S: age distribution of patients in both groups. Table 3S: medical history of patients from two groups. Table 4S: comparison of nutritional risk screening scores between two groups. [file 6627620.f1.docx]

**Supplemental Data**

**Table 1S.** Gender distribution of patients in the two groups [n (%)].

| Gender | EBER-positive (n=13) | | | EBER-negative (n=17) | | χ^2^ | | P values | |
| --- | --- | --- | --- | --- | --- | --- | --- | --- | --- |
| Male | | 7 (53.8) | 9 (52.9) | | 0.002 | | 0.961 | |  |
| Female | | 6 (46.2) | 8 (47.1) | |  |  |  |  |  |

**Table 2S.** Age distribution of patients in both groups [n (%)].

| Age | | EBER-positive (n=13) | EBER-negative (n=17) | | χ^2^ | | | P values |  |
| --- | --- | --- | --- | --- | --- | --- | --- | --- | --- |
| 18-44 | 5 (38.5) | | | 5 (29.4) | | -0.089 | 0.934 | | |
| 45-59 | 2 (15.4) | | | 6 (35.3) | |  |  |  |  |
| ≥60 | 6 (46.2) | | | 6 (35.3) | |  |  |  |  |

**Table 3S.** Medical history of patients from two groups [n (%)].

| Medical history factors | | EBER-positive (n=13) | EBER-negative (n=17) | | χ^2^ | P values |
| --- | --- | --- | --- | --- | --- | --- |
| Autoimmune diseases | 1 (7.7) | | | 3 (17.6) | 0.064 | 0.801 |
| Smoking history | 6 (46.2) | | | 4 (23.5) | 1.697 | 0.193 |
| Drinking history | 2 (15.4) | | | 3 (17.6) | 0.000 | 1.000 |
| Hormones | 0 (0.0) | | | 3 (17.6) | - | 0.238^a^ |
| Immunosuppressants | 0 (0.0) | | | 3 (17.6) | - | 0.238^a^ |
| Infliximab | 0 (0.0) | | | 2 (11.8) | - | 0.492^a^ |

^a^ Fisher exact probability method

**Table 4S.** Comparison of nutritional risk screening scores between the two groups [n (%)].

| Nutritional Risk Screening Score | EBER-positive (n=13) | EBER-negative (n=17) | χ^2^ | P values |
| --- | --- | --- | --- | --- |
| <3 | 3 (23.1) | 6 (35.3) | 0.103 | 0.748 |
| ≥3 | 10 (76.9) | 11 (64.7) |  |  |
